# The Field of Science

**Published:** 1894-11-03

**Authors:** 


### The Field of Science.

By way of showing their gratitude to the physicians and students of the College of Medicine in Hamburg for their devoted services during the cholera epidemic in 1892, the authorities have had a medal of honour designed by the well-known artist, A. Vogel. The medal bears the inscription, "To the helpers in distress, grateful Hamburg." On one side appears Hercules struggling with the hydra, and on the reverse the city arms, encircled by a wreath of thorn and oak leaves.

It is gratifying to our national pride to notice once more that Germany is following our lead with regard to matters hygienic. As the result of an inquiry instituted by the Prussian Minister of Education, the old fashion of slanting handwriting will henceforward be discouraged in the State schools, it having been demonstrated that the sloping writing favours a crooked position of the writer, while the upright, with the elbow separated from the side, encourages him to maintain an erect and straight back.

The treatment of red nose has so long been regarded as one beyond the reach of therapeutics, that it is at least interesting to learn that a line of treatment has at last been suggested, It will surprise no one to hear that the treatment suggested is the systematic application of the galvanic current. Both electrodes, we are told, must be applied to the nose and kept in continual motion, the strength of current being adapted to the powers of endurance of the patient. The application is followed by an intense redness of the skin, which may continue for as long as two days, and the treatment must be continued at intervals of a few days. The method requires patience. As many as thirty sittings may be necessary, and of course a long purse and the services of a specialist are essential. A most natural conjunction.

Much has been written on the subject of the innocuousness of the air of sewers, the researches of bacteriologists being in direct contradiction to the practical experience of the public health officers. The association between typhoid and defective plumbing is so popular in acceptance that the investigations of Professor Aleisi, showing by scientific data, however incomplete, the correctness of our long-cherished tradition, are of considerable interest. This observer confined rats, guinea-pigs, and rabbits in boxes with perforated bottoms, placed over open privies, cesspools, and receptacles which received the evacuations of the animals themselves. Notwithstanding that they continued to eat well the animals gradually showed signs of failing health. In this condition small doses of the typhoid bacillus were injected, an operation which was followed by death in all cases, with all the characteristic post-mortem signs of typhoid. Similar inoculation of control animals produced no such consequences. Arguing from analogy, in the case of human beings, we must conclude that persons exposed to sewer-gas have their general bodily resistance to disease lowered to such an extent that they fall an easy prey to the attack of the typhoid bacillus, should it happen to be present in the water, milk, or other article of food.

From Troy, N. Y., we hear a sensational account of a post-mortem examination on the body of an habitual drunkard. The old man appears to have lived till the age of sixty, in spite of his addiction to spirits. The medical man who was performing the examination was prepared, so runs the tale, to find an odour of alcohol within the cranial cavity. This not only proved to be the case, but on applying a match to the surface of the brain a blue flame enveloped the convolutions! Such a tale is calculated to be impressive from the temperance platform, but can have about as much foundation in fact as has the now exploded theory of the spontaneous combustion of drunkards.

The effect of unhealthy dwellings upon children has lately been carefully studied by a Leipsic doctor. He selected for his observations foster children in different parts of the town, all under one year, and has watched them for some considerable time. Dr. Plant examined 43 homes with foster children, one child in each home, and recorded their size, ventilation, state of cleanliness, position as to the sun, &c., and of these 43 homes, eight were very satisfactory, considering they were workmen's homes; 12 were cleanly, had sufficient air, room, and light, and did not greatly offend against the general sanitary laws; eight were fairly good, having room, light, and air about as those just mentioned, but being deficient as regards dampness, uncleanliness, position, &c.; and finally, 15 were poor in all or several respects. The foster children were examined in the homes, and classified as good, middling, and bad, in regard to their state of nourishment. The first class had good muscles, firm bones, and healthy complexions, and had no signs of past or pending constitutional ailments. The second class was pale and rather weak, showing signs of past or existing slight rachitis, but without the more severe nourishment diseases. The third class was tuberculous or short of blood, or the children had rachitis, were exceedingly pale and thin, showing signs of frequent digestive complaints. Of these children 33 were watched for six months, the remainder for a year. The 43 children having been classified in a table dealing with the two questions, milk and dwellings, the following result is arrived at: Good food and good dwellings give, as a rule, healthy children; poor food and poor dwellings invariably miserable children. Is the food poor, but the dwellings good, the children keep healthier and stronger than when the opposite is the case. Are the children, however, sickly and weak to begin with, both poor food and bad dwellings have always a destructive effect, even if only one of these detrimental conditions exists, and the evil effect cannot be counteracted, even if the other condition be ever so favourable. The outcome of Dr. Plant's investigations is consequently this, that both good food and good dwellings are most essential for infants, but that if only one of these two can be procured good dwellings must be given the first place. This is no doubt due to the fact that the influence of the milk varies with the weather and the seasons, whilst the evil effect of bad dwellings is incessantly at work.

